# Assessing User Transparency with Muscle Synergies during Exoskeleton-Assisted Movements: A Pilot Study on the LIGHTarm Device for Neurorehabilitation

**DOI:** 10.1155/2018/7647562

**Published:** 2018-06-03

**Authors:** Andrea Chiavenna, Alessandro Scano, Matteo Malosio, Lorenzo Molinari Tosatti, Franco Molteni

**Affiliations:** ^1^Institute of Industrial Technologies and Automation, National Research Council, Milan, Italy; ^2^Rehabilitation Presidium of Valduce Hospital Villa Beretta, Lecco, Italy

## Abstract

Exoskeleton devices for upper limb neurorehabilitation are one of the most exploited solutions for the recovery of lost motor functions. By providing weight support, passively compensated exoskeletons allow patients to experience upper limb training. Transparency is a desirable feature of exoskeletons that describes how the device alters free movements or interferes with spontaneous muscle patterns. A pilot study on healthy subjects was conducted to evaluate the feasibility of assessing transparency in the framework of muscle synergies. For such purpose, the LIGHTarm exoskeleton prototype was used. LIGHTarm provides gravity support to the upper limb during the execution of movements in the tridimensional workspace. Surface electromyography was acquired during the execution of three daily life movements (reaching, hand-to-mouth, and hand-to-nape) in three different conditions: free movement, exoskeleton-assisted (without gravity compensation), and exoskeleton-assisted (with gravity compensation) on healthy people. Preliminary results suggest that the muscle synergy framework may provide valuable assessment of user transparency and weight support features of devices aimed at rehabilitation.

## 1. Introduction

About 15 millions of people experience a stroke every year worldwide [1], and up to 85% of the survivors suffer from limitations in the activities of daily living (ADLs) because of upper limb motor impairment [2–4]. There are several approaches in rehabilitation practice to reduce motor impairment and to improve upper limb functionality after stroke. The need of containing costs, time, and resources devoted to physical and occupational therapy after injury represents an opportunity for cost-effective and easy-to-use devices that can take over some of the supervisory functions of therapists. In the last decades, robotic rehabilitation has attested as a valuable approach able to provide high-intensity training and increase patient motivation, by assisting motor training [5, 6]. However, one of the main issues in hemiparesis following the stroke event is the lack of strength and motor coordination. Poor motor output of the shoulder joint prevents also the recovery of the distal joints, as they are not adequately stimulated due to the impossibility to reach the target of the task and produce purposeful interaction with the environment. Assistive devices can promote rehabilitation of reaching movements toward an object, provide assist-as-needed motion paradigms [7, 8], or offer different levels of engagement for the user [9]. In the literature, it was demonstrated that robotic-based rehabilitation protocols and conventional therapy induce comparable, positive effects on patients [10]. The main advantages of robot and assistive devices are performing high therapy doses [11] and provide semi-independent movement, which has been shown to increase motivation [12, 13]. Furthermore, robots specifically designed for home rehabilitation allow the chance to continue the rehabilitation in domestic environment. Main drawbacks are high initial costs and the need of an external operator for patients' supervision [10]. To reduce the high cost issue, and to reduce the weight of the system, some devices without actuators have been developed, the so-called “passive exoskeletons” [14]. Passive devices rely on springs and counterweights to generate assistive torques. The efficacy of passive devices and assisted training in general is a matter of debate. However, in the literature, similar therapy outcomes were found when comparing actuated and not actuated robots [15]. In medium/high functionality patients especially, therapies based on active and passive exoskeletons induced comparable improvements on upper limb function [16–18].

One of the main advantages of exoskeleton devices is the possibility to move freely in the workspace and, at the same time, to allow reaching and manipulating objects with the hand. Some studies underlined the importance of exploration of the workspace as a key factor for functional recovery [16]. For this reason, since arm elevation is one of the major issues for workspace exploration, an antigravity support may be needed. Furthermore, when a high muscle activation is required for completing a task, patients may show abnormal muscle patterns, such as the flexion synergy, with remarkable effects on the kinematic of the movement [18].

Besides gravity support, another desired feature for exoskeletons is transparency, or backdrivability. The backdriving torque can be defined as the amount of torque *T* that a human must apply to the robotic joint in order to perform a user-driven movement. Perfect backdrivability is achieved if *T* = 0 in all conditions [19]; in such a case, the free movement torque is equal to the torque produced while wearing the device, and no additional muscular work is needed to move the limbs. Transparency can be reduced either by high inertia or low joint backdrivability, caused by frictions or mechanical transmission, by specific configurations of the device links such as elbow singularity, occurring, for example, when elbow joint is completely extended and the upper arm and the forearm segments are aligned [14, 20, 21]. Transparency is a desirable feature, since a high-transparent device does not interfere with the process of motor learning, allowing patients to experience the effort-error relationship typical of motor-learning processes [22]. However, in order to be helpful, devices must provide assistance, and in such cases, transparency has to be reduced. Few studies in the literature investigated the concept of transparency in the framework of a user-centered perspective, being the balance between high transparency and assistance crucial in the process of motor relearning [23–25].

The framework analysis based on muscle synergies might be a valuable tool for investigating how, and at which extent, the device alters motor modules and affects transparency. Muscle synergies are defined as a spatial-coordinated recruitment of a group of muscles elicited by a shared neural command or specific activation waveforms [26]. The muscle synergy framework was developed to analyze the hypothesis that the central nervous system (CNS) organizes modularly to simplify the production of motor outputs. In such a view, muscle synergies represent a small subset of stored activation patterns on which the CNS can rely on to execute a large number of different movements [27]. This result can be achieved because muscle synergies can be tuned in time and magnitude [28, 29].

Muscle synergies have been widely employed in studies on healthy people to investigate motor control during ADL such as upper-limb reaching or walking [28–31]. While several studies investigated the coupling between muscle synergies with robot control algorithms [32], only a few works have analyzed the interaction with a rehabilitation device, despite the potential of the method in quantifying several aspects such as weight support, muscle pattern alteration, and global device transparency. A passive weight support device was used to investigate the effects of different levels of gravity compensation on muscle synergies on a set of reaching movements, concluding that spatial synergies are only slightly altered and temporal components decrease proportionally to the level of support [33]. A recent study employed EMG and muscle synergies for a detailed analysis of an upper limb exoskeleton in various interaction conditions [34]. Other studies instead analyzed the effects of a planar end-effector training on muscle synergies in acute poststroke patients [35].

In previous works, the LIGHTarm exoskeleton device was presented [36] and characterized in a preliminary study while holding static postures and performing dynamic movements [37]. The study suggested cautious good results for gravity compensation; an almost unchanged EMG signal was found when the device was not gravity-compensated, while reduced EMG activity was observed when compensated. A more refined EMG analysis might evaluate transparency features as a modification of spatial and temporal components of muscle synergies underlying movement. To authors' knowledge, very rarely EMG-based methods were adopted to estimate transparency of robot devices. In this paper, a method for the quantitative evaluation based on muscle synergies was carried out to explore the possibility of evaluating user transparency, that is, if the interaction with a passive exoskeleton (the LIGHTarm device) alters muscle synergies spatial and temporal composition, and at what extent, in respect to free movements.

## 2. Materials and Methods

### 2.1. Participants

Three healthy subjects were enrolled in this study (Table 1). Subjects had no previous experience with the LIGHTarm device. Each subject signed a written informed consent form before inclusion in the study. The study was conducted in compliance with the Declaration of Helsinki.

### 2.2. The LIGHTarm Device

**Figure 1:**
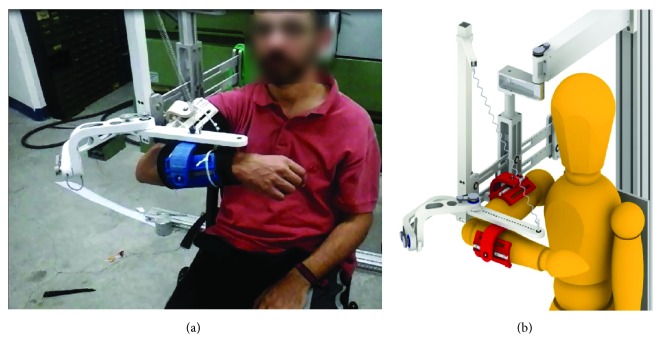
The LIGHTarm exoskeleton: prototype and rendering.

The LIGHTarm device (Figure 1) consists of a hybrid mechanism composed of a serial and a parallel kinematic chains. The architecture was conceived to allow physiological movements of the shoulder joint and avoid singular configurations of the upper limb, especially of the elbow joint. The weight support mechanism was designed as a combination of two separate mechanical elements: a counterweight system supporting the whole arm and a spring-based system supporting the elbow joint. The architecture was conceived to avoid constriction on the shoulder, especially during abduction when a coupled shoulder elevation occurs [14], and therefore preserve the scapulohumeral rhythm, which is a key issue in the exoskeleton design. Thanks to the not-actuated design and simple structure, LIGHTarm can be considered an affordable device. More detailed description of the design of the device can be found in previous works [36, 37].

The experimenters measured subjects' anthropometry of the arm and the forearm and tuned the LIGHTarm so that the shoulder and the elbow of the subjects were aligned with the exoskeleton joints. Then, a proper counterweight was added so that the weight of the device (without limb) was compensated. In this way, the weight of the links anterior to the parallelogram did not influence the execution of movements. After the tuning procedure, the subject was fastened with the strap pads. The arm compensation was chosen as the amount of weight required to maintain the arm raised in the position depicted in Figure 1, tested after the operator had passively raised the arm of the subject being tested. Once weight compensation was defined, the subjects executed all the tasks in one-single session without taking off the device.

### 2.3. Materials and Measures

EMG signals were recorded at a sample frequency = 1000 Hz, with an 8-channel EMG acquisition system (FreeEMG, BTS, Italy) to evaluate muscular activation patterns of the following muscles: deltoids anterior, middle, and posterior, upper trapezius, pectoralis major, triceps lateral head, biceps brachii caput longum, and brachioradialis of the right limb. Such muscles were chosen since they are mainly involved in upper limb tasks with focus on exploration of the workspace.

Kinematics of the right limb was recorded with a 6-TVC marker-based motion capture system (Smart-D, BTS, Italy). Markers were positioned on C7 and D5 vertebras, acromion, right epicondyle of the elbow, and styloid process of the ulna [38]. The elbow marker was at times not tracked due to the exoskeleton encumbrance. A four-marker cluster, placed on the arm, was used to infer elbow position.

### 2.4. Motor Tasks

**Figure 2:**
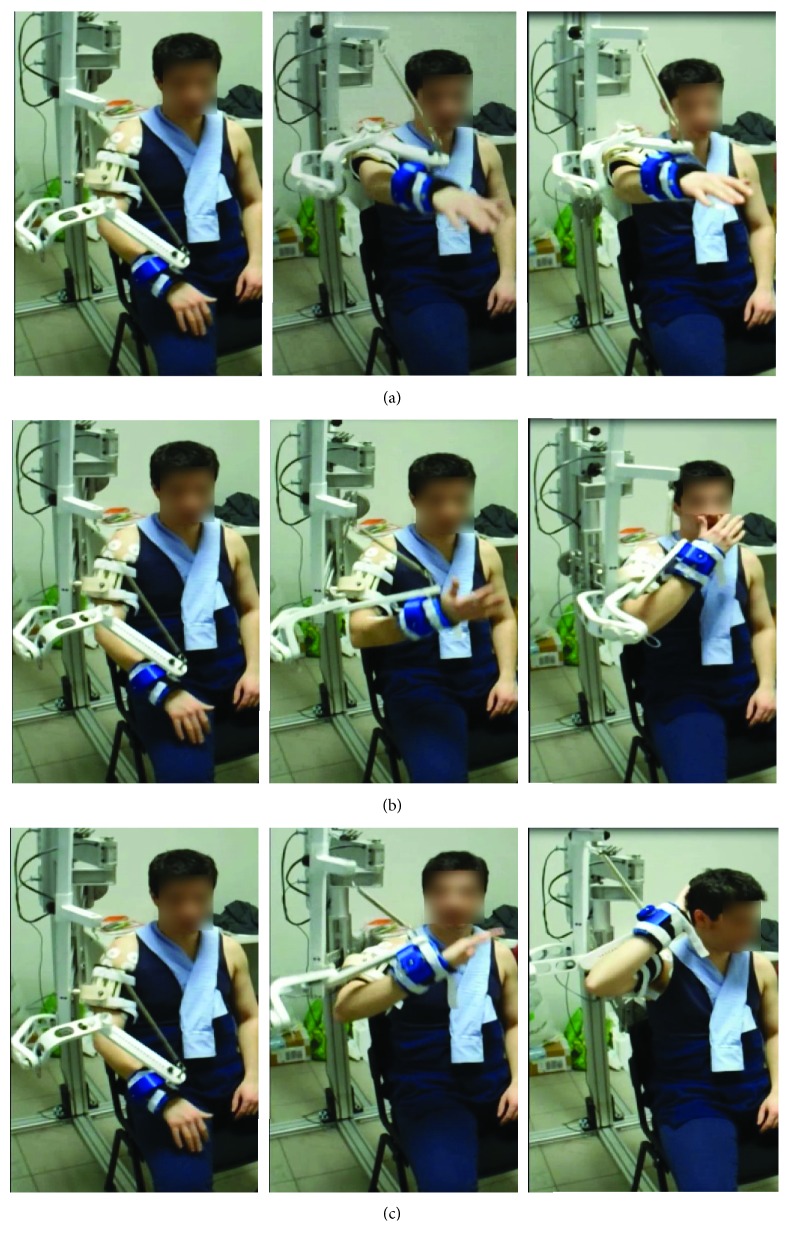
The three motor tasks: (a) reaching, (b) hand-to-mouth, and (c) hand-to-nape.

The tasks selected to evaluate the LIGHTarm were functional movements usually performed in everyday life. The starting position was the same for every movement; the subject was seated on a chair with the hand lying on a cushion positioned on the thigh. The subject performed 12 repetitions of each task at a self-selected speed without pauses between one repetition and the following. The three movements proposed are listed below:

- Reaching against gravity (RCH, Figure 2(a)): from the starting position, the subject raised the arm at 90° of shoulder flexion, 0° of shoulder abduction, and with elbow and the fingers extended.
- Hand-to-mouth (HTM, Figure 2(b)): from the starting position, the subject raised the arm and flexed the elbow to bring the hand to the mouth.
- Hand-to-nape (HTN, Figure 2(c)): from the starting position, the subject raised the arm until the hand was in contact with the nape.

The three tasks were executed in three different conditions: free movement without the exoskeleton (free), with the exoskeleton without arm weight compensation (not compensated), and with the exoskeleton with arm weight compensation (compensated).

### 2.5. Muscle Synergy Extraction

EMG and kinematic data were recorded during each set of 12 repetitions. Then, the first and the last movements were discarded, and only the forward phase of each repetition was considered for synergy extraction. Movement phases were detected through kinematic analysis, applying an automatic phase detector algorithm based on the velocity of vertical coordinate of the wrist marker as a reference for RCH and HTM movements and on the velocity of vertical coordinate of the elbow marker as a reference for the HTN movement. If the elbow marker tracking was not available due to exoskeleton obstruction, the lost frames were reconstructed through the four-marker cluster. Data from retroreflective markers were filtered with a low-pass, 3rd-order Butterworth filter, with cut-off frequency set at 6 Hz.

EMG signals of the eight muscles in the forward phase were filtered (high-pass filtering (50 Hz), full-wave rectification, FIR low-pass filtering (cut-off frequency = 20 Hz) [34]) in obtaining the envelope of the signal. EMG data from each subject and each trial were pooled together in a single-aggregated matrix, and synergies were extracted using the nonnegative matrix factorization (NMF) algorithm [38]. The NMF decomposes the electromyography (EMG) matrix into the product of two matrices, the first one representing time-invariant, spatial-coded synergies (*w*_*i*_), and the second one representing time-variant activation commands for each synergy (*c*_*i*_) [31], as in the following:

(1) $$\begin{array}{c} EMG\left(t\right)=\overset{N}{\underset{i=1}{\sum }}c_{i}w_{i}, \end{array}$$

where for each of the recorded muscles, EMG(*t*) represents the EMG data at time *t* and *N* is the total number of extracted synergies.

The procedure of synergy extraction was performed by pooling together the EMG envelope matrix of each acquisition, including ten repetitions of the motor task for each experimental condition (3 subjects × 3 motor tasks).

The order of the factorization *r* was chosen increasingly from 1 to 8 (maximum number of muscles that characterizes the dimensionality of the problem). For each *r*, the NMF algorithm was applied 100 times in order to avoid local minima, and the repetition accounting for the higher variance of the signal was chosen as the representative of order *r*. The number of synergies was chosen as the minimum *r* explaining at least 0.75 of the total variance of the signal [33].

For representation purposes, authors ordered synergy datasets by matching synergies that have a similar functional role within a specific gesture. For such reasons, synergy datasets were matched at best by considering the Pearson correlation coefficient of the temporal components. After the matching procedure, extracted synergies are naturally matched so that they are at best comparable between experimental conditions.

Then, the dataset of extracted spatial synergies was split into three subdatasets: the first one comprehended the synergies extracted from free movements, the second one including synergies extracted from noncompensated assisted movements, and the third one including compensated movements. A k-means cluster analysis was conducted on each of the three datasets, to identify mean spatial synergies (centroids) for each of the experimental conditions. The order of each clustering was selected by considering a tradeoff between accuracy and synthesis, pondering indexes related to clustering quality such as silhouette and Euclidean distance of synergies from their reference centroid. Finally, each temporal component was coupled to its mean spatial synergy.

### 2.6. Outcome Measures: User Transparency

While several definitions of transparency are given in the literature [24, 25], for passive exoskeletons, the concept of *user transparency* is here introduced. User transparency may be defined as the alteration of motor modules (here modelled as muscle synergies) due to the interaction with an exoskeleton. Alterations may be due to device encumbrance, singular configurations, mechanical locks or couplings, or weight support features. In this paper, it is proposed that user transparency can be assessed in the framework of muscle synergies. For a device that is aimed at producing weight support, transparency may be “decomposed” into two main contributions. At first, a desirable transparency term is related to weight support. As a consequence of LIGHTarm support, the magnitude of temporal components should be reduced, because of the less effort needed to elevate the limb. A second term, instead, deals with the modifications of the spatial synergy composition. It investigates how the motor modules are modified due to the interaction with the device. Since the weight support action should not alter the spatial composition of motor modules underlying movement, preservation of muscle patterns in assisted movements in respect to free ones is considered as an index of the effect of the exoskeleton to preserve unaltered physiological patterns and not interfere with spontaneous EMG activity.

In summary, coordinated muscle patterns can be evaluated by considering the difference in the composition of spatial muscle synergies, while weight support features can be analyzed by considering the magnitude of the temporal component associated to each synergy.

Consequently, in this work, the evaluation of user transparency is split into two components:

1. Mean spatial synergy similarity, investigating if LIGHTarm alters muscle patterns during dynamic motion.
2. Weight support features, investigating if LIGHTarm is effectively reducing the magnitude of the temporal components related to spatial synergies.

In order to quantify pattern alteration, the similarity of mean spatial synergies is considered. The metrics chosen for detecting similarity among mean spatial synergies (centroids) were the dot product, which was already used in previous studies as an indicator of synergy similarity [39–41]. A high dot product value corresponds to a good similarity between the conditions, indicating that the presence of the exoskeleton would not influence synergy composition. Dot product values range from 0 (no similarity) to 1 (perfect similarity). Dot products were calculated between each synergy pair obtained by matching free movements, LIGHTarm-assisted movements in compensated set-up, and LIGHTarm-assisted movements in noncompensated set-up.

For the evaluation of the weight support features, the integral of each mean temporal component (mtc) was calculated as a representative of the magnitude of the activation of each spatial synergy. A reduction in the integral value is evidence of less muscular effort needed to perform the movement. The mtc were calculated for each mean temporal component as follows:

(2) $$\begin{array}{c} mtc=\int_{t_{0}}^{t_{f}}{\bar{c}}_{i}\left(t\right)dt, \end{array}$$

that is, the integral of the mean temporal component associated to each mean spatial synergy.

## 3. Results

### 3.1. Synergy Extraction

**Figure 3:**
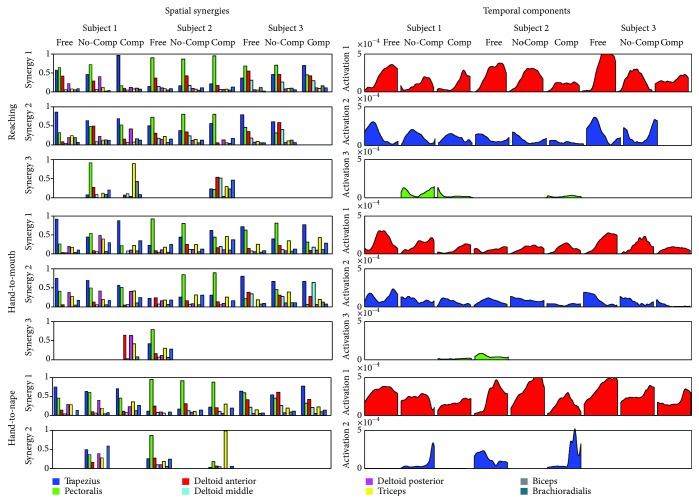
Synergy spatial composition and temporal components. No-Comp = not compensated, Comp = compensated, TP = upper trapezius, PM = pectoralis major, DA = deltoid anterior, DM = deltoid middle, DP = deltoid posterior, TRI = triceps brachii, BIC = biceps brachii, BR = brachioradialis.

Spatial synergy compositions, matched by correlation of temporal components, for each of the considered tasks and subjects, are shown in Figure 3.

### 3.2. Spatial Synergy Alteration

**Figure 4:**
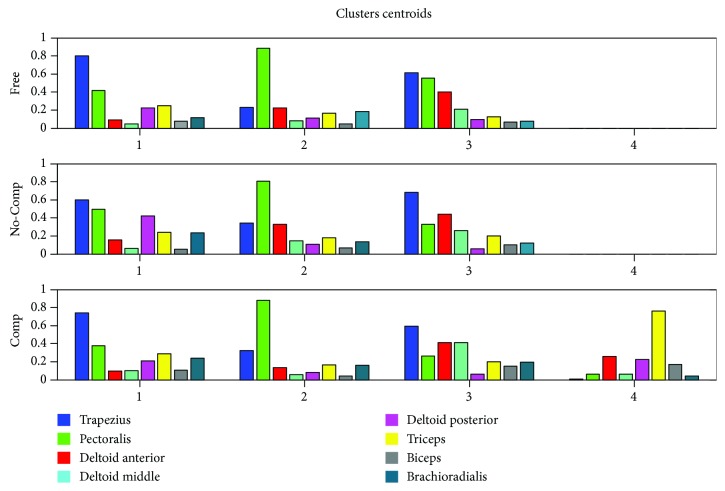
Mean spatial synergies (centroids) for each of the three experimental conditions, matched by similarity. It is possible to notice that the compensated configuration requires the coordination of a spatial synergy, which was not observed in free movements and in not compensated assistance.

Mean spatial synergies, computed with the clustering k-means algorithm, are shown in Figure 4.

Pairwise dot products relative to mean spatial synergy compositions are shown in Table 2.

Dot products between free, not compensated, and compensated movements are always >0.80 for all the pairwise matched mean spatial synergies, indicating that the basic muscle patterns underlying movements are not consistently altered. All the values found are above the range of baseline dot products identified in previous studies in the literature to quantify similarity [39], and therefore, a high (>0.75) or very high (>0.90) similarity [40] is found in this study among mean spatial patterns.

### 3.3. Weight Support Features

**Figure 5:**
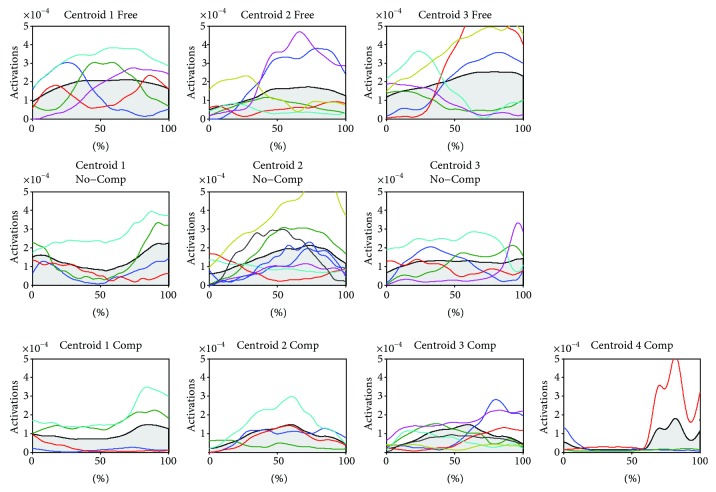
Mean temporal components related to each mean spatial synergy. Free = free movements, No-Comp = not compensated, Comp = compensated.

Table 3 reports the mtc computed for each mean temporal component. For easier visualization, temporal components are graphically reported in Figure 5.

For each mean spatial synergy, the higher mtc value is found in free movements (except centroid 4, which was not needed to describe the dataset in free movements). The mtc in movements performed with LIGHTarm in not compensated set-up indicate that there is a tendency toward a slight reduction of muscle activity. When LIGHTarm was used in the compensated set-up, the mtc decreases consistently.

## 4. Discussion

A detailed review of the insights provided by muscle synergies for the assessment of user transparency is presented in the following sections.

### 4.1. Spatial Synergy Alteration

In comparison to traditional methods for EMG analysis, muscle synergies capture spatial and temporal features that are shared by groups of coactivating muscles, which, according to this framework, are controlled as groups rather than autonomous entities. Consequently, the muscle synergy approach is particularly suited for evaluating pattern alterations induced at the neural level when interacting with a device.

Referring to Table 2, it is possible to say that, averagely, good similarity between synergy composition in the different experimental conditions was found, especially considering the reference values found in the literature (>0.75 high similarity, >0.90 very high similarity) [39, 40]. When the similarity of synergy compositions is above 0.90, the device is not altering the modules underlying movement in a relevant manner. In the specific case of LIGHTarm, when comparing free movement to the ones without weight compensation, high similarity was found when considering the three main spatial patterns underlying the considered daily life gestures. On the contrary, the compensated configuration, which is the one that should be used for rehabilitation for providing full weight support, induces relevant modifications of the mean spatial synergies.

In fact, loss of transparency might be observed in the emergency of new motor modules (centroid 4). While the main modules are in general preserved, all subjects had to rely on some trials on an additional synergy, characterized by abnormal triceps activation. This result can be interpreted in an excessive gravity compensation imposed on the upper limb, as at the end of the range of motion the device was still providing support on the arm, slightly pushing it upwards. Probably, this effect induced triceps compensation, needed to slow the shoulder flexion effect exerted by the exoskeleton. These observations might be valuable for further tuning of the device or for its partial redesign.

While the sample of subjects is too low for proposing statistical analysis for the specific case of LIGHTarm, the explained methodology proposes valuable insights on muscle coordination while interacting with a device and may help in deducing if a generic exoskeleton may induce modifications to the motor modules underlying movement. In hypothesizing to have a wider sample of subjects, the muscle synergy analysis might provide such valuable insights with statistical confirmation—or denial—of the results.

### 4.2. Weight Support Features

For a device like LIGHTarm which is aimed at supporting the weight of the limb, the weight support features are a needed “loss of transparency”; the magnitude of temporal components should be reduced to allow elevating the limb against gravity with less effort. Weight support features can be evaluated by considering the magnitude of the activation profile of each synergy. In the muscle synergy framework, the reduction of magnitude of a module is seen as the reduction of activity of a whole set of muscles responsible for a specific kinematic movement. In case of a decrease of the temporal component integrals, the device is inducing an effect of reduced effort, allowing the subject to elevate the arm with less EMG activity. In the specific case of LIGHTarm, a reduced magnitude is always shown between the not compensated and the compensated configurations. A magnitude difference can also be noticed between the free and not compensated configurations, with decrement of the integral values in most of the trials. Despite the counterweight was specifically set only for the compensation of the weight of the exoskeleton, wearing the device induced an effect on the amount of muscle activation needed to complete the movements. This could be interpreted with a nonhomogenous support in the workspace, with a slight overcompensation of the shoulder when over 90° of shoulder flexion as seen in the RCH and HTN and a little resistance contribution in the lower part of the workspace that require more muscle activation to achieve the target, as seen in HTM.

All the reported results are preliminary and, due to the low number of subjects, are not statistically significant. However, they show how the muscle synergy framework might be valuable for assessing the weight support features of an exoskeleton device.

### 4.3. Implications and Limitations

As proposed in many papers [7, 23], transparency is a key feature that a robotic device should have to provide valuable assistance to patients in rehabilitation. While remarkable efforts have been done in the literature to design devices and controllers for achieving transparency [23, 24, 42], few works have investigated the potential of a modular description of the neuromuscular system in evaluating the properties of a device. In this paper, it is suggested that muscle synergies may be considered for comparing motor modules underlying movement in free movements and robot-assisted ones, providing a user-centered view of the transparency properties of the device. While a few other studies suggested [23] or exploited [33–35] the potential of muscle synergies as outcome variables for assessing human-robot or human-device interaction in the rehabilitation field, even if in a very basic form, here, it is proposed that muscle synergies may represent a very interesting framework for testing transparency.

This study has several limitations. At first, the number of enrolled subjects is low and does not allow providing statistical conclusions over LIGHTarm transparency. However, several aspects that might be observed in the framework of muscle synergies have been considered, and their applicability to other devices has been discussed. Furthermore, authors acknowledge that many features of muscle synergies could be examined in more detail. A study design including a comprehensive exploration of the workspace would elicit a higher variety of motor modules, allowing a detailed mapping of motor module alterations. This would allow providing a more refined mapping of the repertoire of upper limb motor modules when in interaction with the device, rather than simple, task-specific patterns.

Lastly, a new, refined version of LIGHTarm is about to be released and will be tested in future studies, including a larger number of subjects.

## 5. Conclusion

In this work, the concept of transparency was assessed in relation to the interaction with passive exoskeleton devices for rehabilitation. Preliminary assessments were proposed, suggesting the potential of the muscle synergy-based approach for the evaluation of transparency. Such method might lead to the definition of the alteration induced by the device and its weight support features.

## Figures and Tables

**Table 1:** Participants.

ID	Subjects			
	*Age*	*Sex*	*Height*	*Weight*
Subject 1	46	M	181	68
Subject 2	23	M	183	85
Subject 3	29	M	179	78

**Table 2:** Pairwise dot products of the mean spatial synergies (centroids) in the different experimental conditions. Free = free movements, No-Comp = not compensated, Comp = compensated. n.a. = not available data, / = comparison with the same condition.

	Centroid 1			Centroid 2			Centroid 3			Centroid 4		
	Free	No-Comp	Comp	Free	No-Comp	Comp	Free	No-Comp	Comp	Free	No-Comp	Comp
Free	/	0.89	0.92	/	0.94	0.95	/	0.88	0.82	/	n.a.	n.a.
No-Comp	0.89	/	0.87	0.94	/	0.93	0.88	/	0.86	n.a.	/	n.a.
Comp	0.92	0.87	/	0.95	0.93	/	0.82	0.86	/	n.a.	n.a.	/

**Table 3:** mtc values related to each mean spatial synergy. No-Comp = not compensated and Comp = compensated.

	Centroid 1	Centroid 2	Centroid 3	Centroid 4
Free	0.2071	0.1307	0.1854	0
No-Comp	0.1334	0.1208	0.1502	0
Comp	0.0906	0.0548	0.0960	0.0832
